# Reporting quality of abstracts and inconsistencies with full text articles in pediatric orthopedic publications

**DOI:** 10.1186/s41073-023-00135-3

**Published:** 2023-08-23

**Authors:** Sherif Ahmed Kamel, Tamer A. El-Sobky

**Affiliations:** 1 Department of Orthopedic Surgery, Faculty of Medicine, Ain Shams University, Cairo, Egypt; 2 University Hospitals of Leicester NHS Trust, Leicester, UK; 3 Division of Pediatric Orthopedics, Department of Orthopedic Surgery, Faculty of Medicine, Ain Shams University, Cairo, Egypt

**Keywords:** Manuscript title, Journal abstract, Article summary, Academic writing, Scholarly authorship, Orthopedic periodicals

## Abstract

**Background:**

Abstracts should provide a brief yet comprehensive reporting of all components of a manuscript. Inaccurate reporting may mislead readers and impact citation practices. It was our goal to investigate the reporting quality of abstracts of interventional observational studies in three major pediatric orthopedic journals and to analyze any reporting inconsistencies between those abstracts and their corresponding full-text articles.

**Methods:**

We selected a sample of 55 abstracts and their full-text articles published between 2018 and 2022. Included articles were primary therapeutic research investigating the results of treatments or interventions. Abstracts were scrutinized for reporting quality and inconsistencies with their full-text versions with a 22-itemized checklist. The reporting quality of titles was assessed by a 3-items categorical scale.

**Results:**

In 48 (87%) of articles there were abstract reporting inaccuracies related to patient demographics. The study's follow-up and complications were not reported in 21 (38%) of abstracts each. Most common inconsistencies between the abstracts and full-text articles were related to reporting of inclusion or exclusion criteria in 39 (71%) and study correlations in 27 (49%) of articles. Reporting quality of the titles was insufficient in 33 (60%) of articles.

**Conclusions:**

In our study we found low reporting quality of abstracts and noticeable inconsistencies with full-text articles, especially regarding inclusion or exclusion criteria and study correlations. While the current sample is likely not representative of overall pediatric orthopedic literature, we recommend that authors, reviewers, and editors ensure abstracts are reported accurately, ideally following the appropriate reporting guidelines, and that they double check that there are no inconsistencies between abstracts and full text articles. To capture essential study information, journals should also consider increasing abstract word limits.

**Supplementary Information:**

The online version contains supplementary material available at 10.1186/s41073-023-00135-3.

## Background

Academic writing is an invaluable tool for communicating research findings to the scientific community and the wider public. This is critical to the advancement of clinical practice and refinement of consensus guidelines for treatment of various diseases across medical disciplines. The title and abstract section of articles are intended to convey a brief yet comprehensive and systematic reporting of studies objectives, material and methods, results, and conclusions. Ideally, the title and abstract should provide a standalone summary aimed at informing authors about the appropriateness of the article content to their ongoing or upcoming research work, and informing clinicians about the recent trends in management of challenging disorders [1, 2].

Nevertheless, various systematic reviews have found inconsistencies between abstracts and published articles in orthopedic physical therapy [3], spine surgery [4], other surgical [5], and medical [6] disciplines. Additionally, studies found suboptimal reporting quality of abstracts of randomized control trials in relation to reporting guidelines recommendations [7]. Conference abstracts, i.e. preliminary unpublished research, were found to be more prone to such inconsistencies [6, 8]. Without adequate reporting, scientifically sound and innovative research could pass unnoticed, while wrongly reported research could mislead readers and patients [6, 9]. For example, misinterpretation of statistical significance in abstracts can result in overstated or unjustified conclusions. Additionally, studies have demonstrated inaccurate citation practices across various medical and surgical specialties, including different orthopaedic surgery subspecialties [10–14].

Inaccurate citation practices—can misrepresent or even contradict the conclusions or interpretations made in the article that is being cited [10, 15]. Inaccurate citation practices can also occur when authors cite abstracts, without reading the corresponding full-text articles [10, 15, 16], especially when there are inconsistencies between the two. This is particularly important for busy clinicians and for non-open access articles. In such situations the abstract becomes the most practical and occasionally the only available source of information [17].

Therapeutic/interventional studies that investigate surgical or nonsurgical treatment as pre/post observational case series are common occurrence in orthopedic surgery literature in general and pediatric orthopedic in specific [18]. The primary objective of this study was to investigate the reporting quality of abstracts of observational studies in three major pediatric orthopedic journals against an itemized checklist. The secondary objective was to analyze any reporting inconsistencies between the abstracts and their corresponding full-text articles.

## Material and methods

We included original articles from the top three pediatric orthopedic society journals: 1) Journal of Pediatric Orthopedics, the official journal of the Pediatric Orthopaedic Society of North America; 2) Journal of Pediatric Orthopaedics part B, the official journal of International Federation of Paediatric Orthopaedic Societies; and 3) Journal of Children's Orthopaedics, the official journal of the European Paediatric Orthopaedic Society. To be included articles needed to be primary therapeutic research in pediatric orthopedics investigating the results of a treatment or an intervention, prospective or retrospective observational study. We excluded prognostic (natural history), diagnostic and economic studies, systematic reviews and meta-analysis, randomized control trials, case reports, editorials and other non-original research, and those whose full-text was irretrievable. The second author T.A.E searched the included journals’ websites for articles. We selected a convenience sample by including first eligible article per journal issue.

### Screening and data analysis

To achieve the primary objective, we determined the abstract reporting quality using an itemized checklist (Additional file 1). The checklist was developed by us the authors and was based on (STROBE) guidelines for reporting of observational studies https://www.strobe-statement.org/. All items of the checklist had a dichotomous manner, i.e. item was present or absent. To achieve the secondary objective, we compared the information presented in each abstract with information presented in its corresponding full-text article. Thus, we retrieved all full-text articles and subjected them to free and in depth reading with the aim of; 1) identifying missing or inaccurately reported information in the abstract and 2) recording inconsistency/disagreement between information presented in the abstract and that presented in the full-text. The first author S.A.K performed analysis and revision of half the study sample, while the second author T.A.E performed the above screening, analysis, and revision of the whole sample, i.e. final 55 included articles. Disagreements between authors were resolved by joint meetings. We used descriptive statistics based on the total number of included studies.

### Checklist items and definitions

Checklist consisted of 22 items, of which two asked about the title, 13 about quality of the abstracts, and 7 about inconsistencies between the abstracts and full texts. Definitions and items they refer to are presented below. We considered information when it was either explicitly or contextually implied.

#### Title

- Insufficient title (Question 1 of the checklist): we considered a title insufficient if one of the following items was missing; population, pathology, intervention, or follow-up period mentioned descriptively or numerically. This was irrespective of whether the title was in an indicative/informative or interrogative (question) format [1, 2].
- Misleading title (Question 2 of the checklist): we considered a title misleading when two or more of the above-mentioned items were missing or had discordant information [1, 2]. For example, the title indicates or implies long-term follow-up while in fact it is not, or title indicates cerebral palsy children while in fact study population comprises other neuromuscular disorders.

#### Abstract

- Reporting quality of abstract refers to the fulfilment of a certain reporting item scenario of the abstract per se and irrespective of the information present in its corresponding full-text article [1, 6, 7, 19, 20].
- Missing information: we identified missing as either essential item/information reported in the full-text article and not in the abstract or vice versa [3–6].
- Discrepant information: refers to discrepancies or disagreements between information reported in abstracts and that reported in its corresponding full-text article. Examples are; exaggerated or excessively generalized conclusions which are not substantiated by the results presented in the full-text article or discrepancies between sample size, gender distribution, *P* values in the abstract and in the full-text [3–6].
- Essential item of abstract: we identified essential information/items of the abstract as obligate reporting information the absence of which would compromise the reporting quality of the abstract, irrespective of the information being reported in the full-text article or not. Examples are; reporting at least one study objective, reporting study population characteristics, type of intervention/treatment, and so forth [1, 6, 7, 19, 20].
- Non-essential item of abstract: we identified non-essential information/items of the abstract as an occasionally obligate reporting information depending on the study context and settings, and the absence of which would not necessary compromise the reporting quality of the abstract. Vetting the full-text article can determine the applicability of reporting non-essential information to a particular abstract. Examples are; presence of a second or third study objective that was not reported in the abstract, yet reported in the full-text article or presence of a second or third outcome measure that was not reported in the abstract, yet reported in the full-text article, and so forth [1, 4, 7].

Definitions and virtual examples of what constitutes *accurately* reported items in abstracts are shown in (Table 1), while study’s raw data with explanatory comment is publicly made available (https://doi.org/10.5281/zenodo.7616147).

**Table 1:** Accurately reported items in abstracts: Definitions and virtual examples^a^

Abstract item	Definition of accurate reporting	Virtual examples
Primary objective	Accurate reporting entails systematic & brief reporting of the study design/type, intervention, outcome measures used, disease & patient population characterization e.g. child/adult, follow-up period^b^	• the objective of this study was to retrospectively evaluate the intermediate-term clinical and radiological outcomes of salvage femoral valgization osteotomy in non-ambulatory cerebral palsy children with late presenting neglected hip dislocation • the objective of this study is to prospectively evaluate the short-term efficiency of an intensive physical rehabilitation regime following multi-level orthopaedic surgery for ambulatory cerebral palsy children and adolescents
Inclusion/Exclusion criteria	Accurate reporting entails systematic & brief reporting of inclusion/exclusion criteria respecting all “relevant/applicable” details of patient disease/population eligibility criteria, intervention-related criteria, resources or follow-up plan/period etc	*Included* patients needed to be children with spastic cerebral palsy and not previously subjected to a bony hip procedure. We *excluded* adolescents, other non-spastic forms of cerebral palsy, those previously subjected to a bony hip procedure or botulinum toxin injection within the past year proceeding index operation
Correlations of variable in results	Accurate reporting entails clear identification of correlated variables & result of correlation e.g. significant/insignificant or significant positive/negative etc.;	The total and individual subscores (X) & (Y) score of Health-related Quality of Life showed a statistically significant improvement in group 1 patients *P* = < .05. Individual subscores (Z) improved yet didn’t reach statistical significance in the same group. Total and all subscores of Health-related Quality of Life scores didn’t show any statistical significance in group 2 patients. All results at mean follow-up of two years
Study conclusions or key points^c^	Accurate reporting entails interpreting the study results within its specific clinical context/settings and limitations. This should be performed in view of the stated study objectives	There is credible evidence to support the use of the current manipulative casting technique for idiopathic congenital clubfeet (< 3 years) who have not been previously operated, on the short-term
Implications of study results	Accurate reporting entails delineating the potential impact or applicability of the results beyond the specific study settings. For example, applicability of the employed intervention to other disease populations or other sub-types of the studied disease etc	The satisfactory results of salvage hip procedures in neglected hip dislocation of non-ambulatory cerebral palsy children may be investigated in hips of other non-ambulatory neuromuscular disorders

^a^Some essential items of abstract as follow-up period, prospective versus retrospective design etc., may not have been reported under study objectives yet were reported under other sections of the abstract e.g. methods. Because an article’s abstract represents a conceptual continuum, the previous situations are still considered accurate reporting; ^b^Occasionally, patient population is implied e.g. congenital implies children etc., ^c^Simple summarizing of study results –without interpretation- was not considered as conclusions/key points

## Results

We included a total of 55 articles (and their abstracts) published between 2018 and 2022. Twenty articles (36%) belonged to Journal of Pediatric Orthopedics, 18 (33%) to Journal of Pediatric Orthopaedics part B, and 17 (31%) to Journal of Children's Orthopaedics. Thirty seven abstracts (67%) were structured and 18 (33%) were non-structured. The included articles covered the following pediatric orthopedic themes; trauma, spine deformities, cerebral palsy, congenital/developmental, knee and foot deformities, adolescent hip disorders, osteoarticular infections, and sports injuries. The list of included articles is shown in (Additional file 1).

**Fig. 1:**
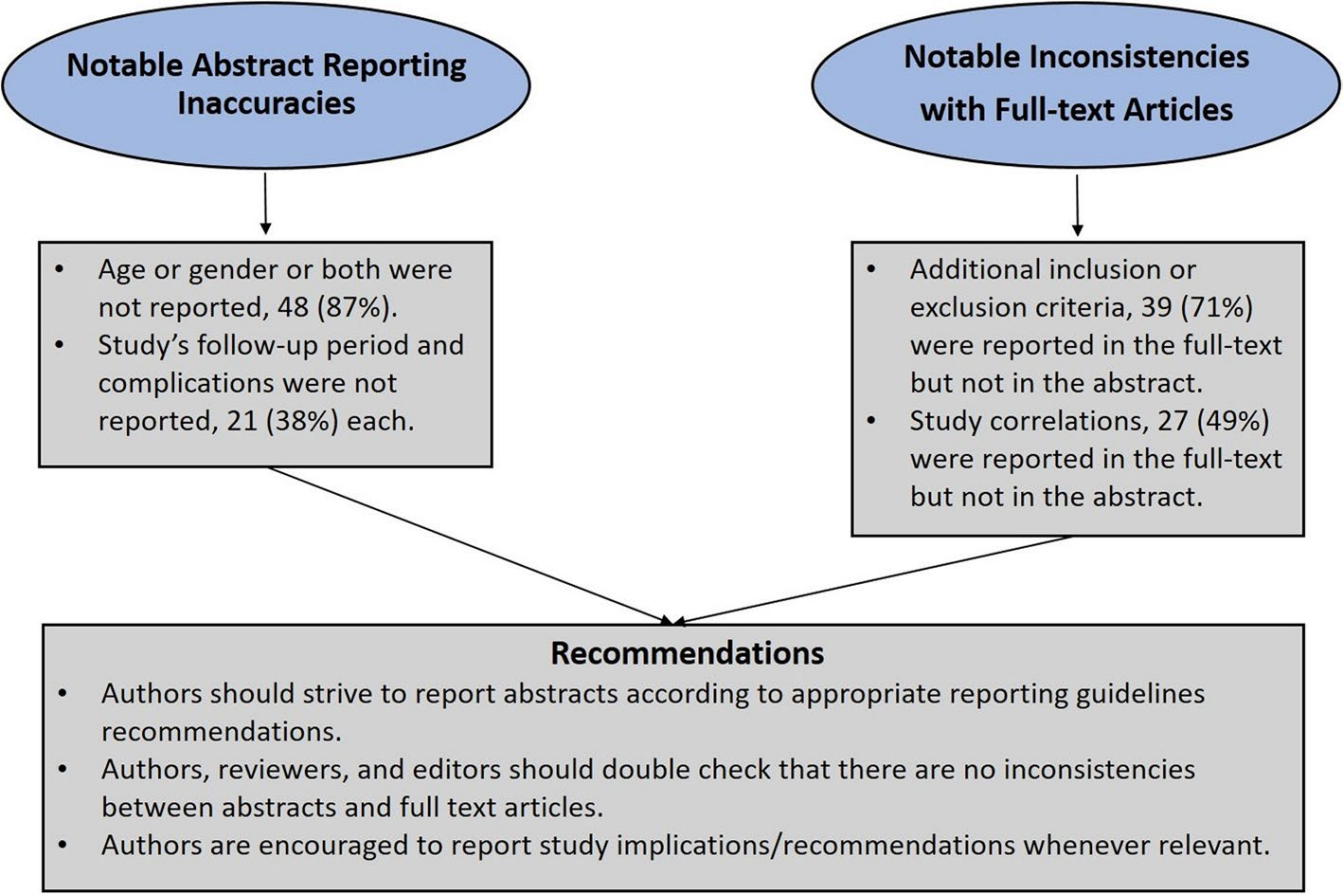
Graphical abstract. Reporting quality of abstracts and inconsistencies with full-text in pediatric orthopedic publications

Quality or accuracy of reported items in titles and abstracts of included articles is shown in (Table 2) and inconsistencies between abstracts and full-text articles in (Table 3). In 48 (87%) of abstracts either age or gender or both were not reported, 21 (38%) of abstracts did not report the study’s follow-up period, and 21 (38%) did not report the study’s complications. Reporting inaccuracies were noted regarding study objectives and inclusion or exclusion criteria. However, there were inconsistencies between the abstract and full-text with respect to additional inclusion or exclusion criteria and study correlations that were reported in the full-text yet not in the abstract, in 39 (71%) and 27 (49%) of articles respectively. Study’s raw data with explanatory comments are provided (10.5281/zenodo.7616147), (Fig. 1).

**Table 2:** Quality or accuracy of reported items in titles and abstracts of 55 pediatric orthopedic articles: descriptive statistics

Reported item	No. of articles (%)	
	Yes	No
Is the title misleading?^a^	*0 (0)*	55 (100)
Is the title insufficient?^b^	*33 (60)*	*22 (40)*
Have objectives been accurately reported in the abstract?	36 (65)	19 (35)
Have age and gender been reported in the abstract?	7 (13)	48 (87)
Has population size been reported in the abstract?	50 (91)	5 (9)
Has disease stage/subtype been reported in the abstract?	50 (91)	5 (9)
Have intervention(s) been specified/reported in the abstract?	52 (94)	3 (6)
Have inclusion and exclusion criteria been reported in the abstract?	37 (67)	18 (33)
Have outcome measures been reported in the abstract?	53 (96)	2 (4)
Has follow-up period been reported in the abstract?	34 (62)	21 (38)
Have all study correlations pertaining to all outcome measures “mentioned” in the abstract been reported in the abstract?	47 (85)	8 (15)
Have complications been reported in the abstract?	34 (62)	21 (38)
Have study conclusions or key points been reported in the abstract?	51 (93)	4 (7)
Have study implications been reported in the abstract?^c^	7 (13)	48 (87)
Have study recommendations been reported in the abstract?^c^	8 (14)	47 (86)

^a^We considered a title misleading when two or more of the following items were missing; population, intervention, pathology, and follow-up period or had discordant information, ^b^We considered a title insufficient if one of the previously mentioned items were missing. This was irrespective of whether the title was in an affirmative or question format, ^c^Whenever applicable or relevant as per study settings

**Table 3:** Inconsistencies between abstracts and full-text articles (*n* = 55)*

Type of inconsistency	No. of articles (%)	
	Yes	No
Have any secondary (additional) study objectives been reported in the full-text but not in the abstract?	12 (22)	43 (78)
Are there any numerical discrepancies between the patient and disease demographics reported in the abstract and those reported in the full-text?	6 (11)	49 (89)
Have any additional inclusion or exclusion criteria been reported in the full-text but not in the abstract?	39 (71)	16 (29)
Have any secondary (additional) outcome measures been reported in the full-text but not in the abstract?	18 (33)	37 (67)
Have any additional study correlations been reported in the full-text but not in the abstract?	27 (49)	28 (51)
Are study conclusions reported in the abstract fully justified by the results in the full-text? That is, not exaggerated or excessively generalized? **(*n* = 51)**^a^	42/51 (82)	9/51 (18)
Are study implications reported in the abstract relevant or applicable as per information present in the full-text? **(*n* = 7)**^b^	7/7 (100)	0 (0)

^*^Missing information refers to information missing in either the abstract or the full-text i.e., could be present in the abstract and missing from full-text article or vice versa, ^a^*n,* refers to the denominator or number of articles that accurately fulfilled the debated item in the first place, in 4 articles it was (NA), non-applicable; ^b^in 48 articles it was (NA), non-applicable

## Discussion

In our study, which analyzed abstract reporting inaccuracies and inconsistencies with full-texts in 55 articles from three high-ranking journals in pediatric orthopedic subspecialties, we found abstract reporting inaccuracies and inconsistencies with full-texts. Specifically most common were abstract reporting inaccuracies related to patient demographics, study's follow-up, and complications. Whereas the most common inconsistencies between the abstracts and full-text articles were related to reporting of inclusion or exclusion criteria and study correlations. Our results are in alignment with results reported across diverse medical disciplines [4, 7, 21, 22].

Readers have been cautioned against depending on abstracts as sole source of information, and citing studies based solely on abstracts [10, 15, 23]. Although this practice violates the perquisites of research accuracy and integrity, it is difficult to estimate its true prevalence. It may even be more difficult to prevent such practices. Efforts to fix the former problem should go in-parallel with efforts to improve early career authors’ academic writing skills [9] and journal editorial and peer review scrutiny [24, 25]. Relevantly, receiving peer reviewer training or mentoring and adherence to journals’ review report checklist can aid early career authors in improving the reporting quality of submitted manuscripts [26]. Artificial intelligence may help editors and peer reviewers in detecting basic yet important reporting errors of submitted manuscripts or abstracts [27]. Nevertheless, vetting a manuscript’s scientific quality, completeness of the methodology, and validity of conclusions is more appropriately tasked with the peer reviewers themselves.

The title is an important yet under-researched element of any scholarly article. Rather than reading the abstract, busy readers may rely totally on screening article titles to identify a specific topic. There is no consensus on what constitutes an ideal title of a scholarly article. Broadly, an informative title should be chosen so as to reflect all main components of a study. However, the specific discipline, speciality, research type, title structure and format as indicative versus interrogative, and article dissemination/impact are factors that must be taken into account when formulating an optimal title [28]. For consensus and research purposes we adopted a specific definition of what constitutes a *sufficient* or *standalone* title suited for interventional pre/post studies. In that regard, we included follow-up period as one of the essential reporting item of the title because our included articles dealt with assessment of effects of therapy/treatment on outcomes. The current study did not show any misleading titles. Nevertheless, 60% of the titles were graded insufficient as per study definitions. This was overwhelmingly due failure to report the follow-up period neither descriptively nor numerically in the title.

This study provided a reasonable sample of pediatric orthopedic literature published in highly-sought journals in this subspecialty. In addition to assessing abstract reporting quality per se, we correlated between abstract and full-text reporting. This allowed us to uncover further anomalies of abstract reporting. The majority of articles had no author overlap (one or more authors in common). For articles that had author overlap, it was mostly not possible to verify the author's role in manuscript writing due to absence of *author contribution statement* in most of the included articles. For articles that provided such statement, one author played a *fundamental* role in writing of two articles/manuscripts. Therefore, we assume that author overlap did not influence our results fundamentally.

### Study limitations

Our study had several limitations. We did not cover a representative sample of all orthopedic journals. We also did not perform a formal quantitative or qualitative statistical sub-analysis of the unreported or inaccurately reported items in the abstract or full-text which was beyond the study objectives. However, we recorded explanatory comments in the study’s raw data file for a deeper look. For instance, some study correlations that were not reported in the abstract yet were in the full—text proved to be highly supportive of the final conclusions in the abstract, while other instances of unreported/missing correlations in the abstract proved to be of secondary importance to the final conclusions. We did not correlate the abstract reporting quality with article or journal metrics, or characteristics of authors. The recording of some checklist items, as study implications, possessed a degree of subjectivity and may be linked to an author’s or assessor’s academic and clinical experience. This may have introduced recording bias. We did not implement a typical dual review or double screening in the search and screening stage of abstracts/titles. The fact that we selected a convenience sample and not an exhaustive one, mitigates this limitation.

## Conclusions

In our study we found low reporting quality of abstracts and noticeable inconsistencies with full-text articles, especially regarding patient demographics, inclusion or exclusion criteria, and study correlations. Our study has shown that more work remains to improve the reporting of abstracts in pediatric orthopaedic literature. While the current sample is likely not representative of overall pediatric orthopedic literature, authors, reviewers, and editors should use relevant reporting guidelines, strive to report at least the items mentioned there, and double check that there are no inconsistencies between abstracts and full text articles. To capture essential study information, journals should also consider easing restrictions on abstract word counts.

### Supplementary Information

**Additional file 1.**  Itemized checklist for assessment of reporting quality or accuracy of abstracts and inconsistencies with full-text articles. And List of final 55 pediatric orthopedic articles included in the analysis of this review.

## Data Availability

The raw data is available at Zenodo repository at: https://doi.org/10.5281/zenodo.7616147.
