# Supplementary material for: Reporting quality of abstracts and inconsistencies with full text articles in pediatric orthopedic publications
Source: Res Integr Peer Rev. 2023 Aug 23;8:11. doi: 10.1186/s41073-023-00135-3 (PMC10463470; doi:10.1186/s41073-023-00135-3)
Supplement: Supplementary file 1 — Additional file 1. Itemized checklist for assessment of reporting quality or accuracy of abstracts and inconsistencies with full-text articles. And List of final 55 pediatric orthopedic articles included in the analysis of this review. [file 41073_2023_135_MOESM1_ESM.pdf]

Reporting Quality of Abstracts and Inconsistencies With Full Text Articles in Pediatric Orthopedic Publications

*Sherif A. Kamel [1, 2], Tamer A. El-Sobky [3]**

[1] Department of Orthopedic Surgery, Faculty of Medicine, Ain Shams University, Cairo, Egypt

[2] University Hospitals of Leicester NHS Trust, Leicester, United Kingdom

[3] Department of Orthopedic Surgery, Faculty of Medicine, Ain Shams University, Cairo, Egypt

*Corresponding author: tamer.ahmed@med.asu.edu.eg

Sherif Ahmed Kamel: sherif_farid@med.asu.edu.eg

Tables of contents

*Itemized checklist for assessment of reporting quality/accuracy of abstracts and inconsistencies with full-texts articles**

Article ID: Journal:

Article Title:

Reporting item description	Yes	No
1) Title was misleading?		
2) Title was insufficient?		
3) Have objectives been accurately reported in the abstract?		
4) Have age and gender been reported in the abstract?		
5) Has population size been reported in the abstract?		
6) Has disease stage/subtype been reported in the abstract?		
7) Have intervention(s) been specified/reported in the abstract?		
8) Have inclusion and exclusion criteria been reported in the abstract?		
9) Have outcome measures been reported in the abstract?		
10) Has follow-up period been reported in the abstract?		
11) Have all study correlations pertaining to all outcome measures “mentioned” in the abstract been reported in the abstract?		
12) Have complications been reported in the abstract?		
13) Have study conclusions or key points been reported in the abstract?		
14) Have study implications been reported in the abstract? ±		
15) Have study recommendations been reported in the abstract? ±		
16) Have any secondary (additional) study objectives been reported in the full-text but not in the abstract?		
17) Are there any numerical discrepancies between the patient and disease demographics reported in the abstract and those reported in the full-text?		

18)	Have any additional inclusion or exclusion criteria been reported in the full-text but not in the abstract?
19)	Have any secondary (additional) outcome measures been reported in the full-text but not in the abstract?
20)	Have any additional study correlations been reported in the full-text but not in the abstract?
21)	Are study conclusions reported in the abstract fully justified by the results in the full-text? That is, not exaggerated or excessively generalized? **(n=51)**
22)	Are study implications reported in the abstract relevant or applicable as per information present in the full-text? **(n=7)**

*****Accurately reported items in abstracts: Definitions and virtual examples are provided in Table 1 of the main manuscript file. ± Whenever applicable or relevant as per study settings, The reporting quality/accuracy of abstracts was assessed by items/questions (3 to 15) of the Itemized checklist. The inconsistencies between the abstracts and the full-texts articles was assessed by items/questions (16 to 22) of the Itemized checklist.

List of final Pediatric orthopedic articles included in the analysis of this review (n=55)

1. *Baraka MM, Hefny HM, Thakeb MF, Mahran MA, El Ghazawy AK, Fayyad TA. Morscher's femoral neck lengthening osteotomy through surgical hip dislocation approach for preservation of Perthes and Perthes-like deformities. J Child Orthop. 2022 Feb;16(1):5-18. doi: 10.1177/18632521221080477. Epub 2022 Apr 5. PMID: 35615389; PMCID: PMC9124916.*
2. *Brown C, Kelly BA, Brouillet K, Luhmann SJ. Ogden Type I to III tibial tubercle fractures in skeletally immature patients: is routine anterior compartment fasciotomy of the leg indicated? J Child Orthop. 2021 Dec 1;15(6):515-524. doi: 10.1302/1863-2548.15.210117. PMID: 34987660; PMCID: PMC8670545.*
3. *Hemo Y, Yavor A, Kalish M, Segev E, Wientroub S. Ponseti treated idiopathic clubfoot - outcome predictive factors in the test of time: analysis of 500 feet followed for five to 20 years. J Child Orthop. 2021 Oct 1;15(5):426-432. doi: 10.1302/1863-2548.15.210156. PMID: 34858528; PMCID: PMC8582614.*
4. *Chomiak J, Ošťádal M, Frydrychová M, Dungl P. Lengthening of the ulna by callotasis in children with multiple hereditary exostoses: comparison of methods with and without internal fixation. J Child Orthop. 2021 Aug 20;15(4):378-387. doi: 10.1302/1863-2548.15.210002. PMID: 34476028; PMCID: PMC8381396.*
5. *Kennedy J, Blackburn C, Barrett M, O'Toole P, Moore D. One and done? Outcomes from 3961 patients managed via a virtual fracture clinic pathway for paediatric fractures. J Child Orthop. 2021 Jun 1;15(3):186-193. doi: 10.1302/1863-2548.15.200235. PMID: 34211594; PMCID: PMC8223081.*
6. *Zhou T, Zhang X, Yu X, Bai Y, Chen H, Li J, Li H, Yu Y. Modified method for reconstruction of thumb abduction function in children undergoing surgical treatment of thumb duplication. J Child Orthop. 2021 Apr 19;15(2):97-105. doi: 10.1302/1863-2548.15.200197. PMID: 34040655; PMCID: PMC8138790.*
7. *Aly AS, Abdelhamid Alsabir AR, Fahmy HA, Fayyad TA. Modified oblique high tibial osteotomy with minimal fixation for correction of adolescent tibia vara: a prospective case series study. J Child Orthop. 2021 Feb 1;15(1):6-11. doi: 10.1302/1863-2548.15.200097. PMID: 33643453; PMCID: PMC7907769.*
8. *Ernat JJ, Wimberly RL, Ho CA, Riccio AI. Vascular examination predicts functional outcomes in supracondylar humerus fractures: a prospective study. J Child Orthop. 2020 Dec 1;14(6):495-501. doi: 10.1302/1863-2548.14.200130. PMID: 33343743; PMCID: PMC7740678.*
9. *Yuan Z, Li Y, Hong K, Wu J, Canavese F, Xu H. Poor delineation of labrum and acetabular surface on arthrogram is a predictor of early failure of closed reduction in children aged six to 24 months with developmental dysplasia of the hip. J Child Orthop. 2020 Oct 1;14(5):372-378. doi: 10.1302/1863-2548.14.200132. PMID: 33204344; PMCID: PMC7666794.*
10. *Ramos O, Burke C, Lewis M, Morrison MJ, Paley D, Nelson SC. Modified Langenskiöld procedure for chronic, recurrent, and congenital patellar dislocation. J Child Orthop. 2020 Aug 1;14(4):318-329. doi: 10.1302/1863-2548.14.200044. PMID: 32874366; PMCID: PMC7453167.*
11. *Braito M, Radlwimmer M, Dammerer D, Hofer-Picout P, Wansch J, Biedermann R. Tarsometatarsal bone remodelling after subtalar arthroereisis. J Child Orthop. 2020 Jun 1;14(3):221-229. doi: 10.1302/1863-2548.14.190190. PMID: 32582390; PMCID: PMC7302416.*
12. *Watkins CJ, Yeung CM, Rademacher E, Kramer DE. Percutaneous leverage technique for reduction of radial neck fractures in children: technical tips. J Child Orthop. 2020 Apr 1;14(2):118-124. doi: 10.1302/1863-2548.14.190130. PMID: 32351624; PMCID: PMC7184647.*

13. *Bonnefoy-Mazure A, De Coulon G, Lascombes P, Armand S. Follow-up of walking quality after end of growth in 28 children with bilateral cerebral palsy. J Child Orthop. 2020 Feb 1;14(1):41-49. doi: 10.1302/1863-2548.14.190125. PMID: 32165980; PMCID: PMC7043123.*
14. *Yuan Z, Xu HW, Liu YZ, Li YQ, Li JC, Canavese F. The use of external fixation for the management of acute and chronic Monteggia fractures in children. J Child Orthop. 2019;13(6):551-559. doi:10.1302/1863-2548.13.190115*
15. *Zanardi A, Fortini V, Abati CN, Bettuzzi C, Salvatori G, Prato E, Di Giacinto S, Lampasi M. Standing and walking age in children with idiopathic clubfoot: French physiotherapy versus Ponseti method. J Child Orthop. 2019 Oct 1;13(5):471-477. doi: 10.1302/1863-2548.13.190097. PMID: 31695814; PMCID: PMC6808080.*
16. *Kubo H, Krauspe R, Hufeland M, Lipp C, Ruppert M, Westhoff B, Pilge H. Radiological outcome after treatment of juvenile flatfeet with subtalar arthroereisis: a matched pair analysis of 38 cases comparing neurogenic and non-neurogenic patients. J Child Orthop. 2019 Aug 1;13(4):346-352. doi: 10.1302/1863-2548.13.190046. PMID: 31489039; PMCID: PMC6701442.*
17. *Hedelin H, Larnert P, Hebelka H, Brisby H, Lagerstrand K, Laine T. Innominate Salter osteotomy using resorbable screws: a retrospective case series and presentation of a new concept for fixation. J Child Orthop. 2019 Jun 1;13(3):310-317. doi: 10.1302/1863-2548.13.180195. PMID: 31312271; PMCID: PMC6598047.*
18. *Goodman AD, Walsh DF, Zonfrillo MR, Eberson CP, Cruz AI Jr. Fluoroscopy as Definitive Postreduction Imaging of Pediatric Wrist and Forearm Fractures Is Safe and Saves Time. J Pediatr Orthop. 2020 Jan;40(1):e14-e18. doi: 10.1097/BPO.0000000000001388. PMID: 30973474.*
19. *Chen ZD, Wu J, Lu CW, Zeng WR, Huang ZZ, Lin B. C1-C2 Pedicle Screw Fixation for Pediatric Atlantoaxial Dislocation: Initial Results and Long-term Follow-up. J Pediatr Orthop. 2020 Feb;40(2):65-70. doi: 10.1097/BPO.0000000000001111. PMID: 31923165.*
20. *Price MJ, Tuca M, Nguyen J, Silberman J, Luderowski E, Uppstrom TJ, Green DW. Juvenile Osteochondritis Dissecans of the Trochlea: A Cohort Study of 34 Trochlear Lesions Associated With Sporting Activities That Load the Patellofemoral Joint. J Pediatr Orthop. 2020 Mar;40(3):103-109. doi: 10.1097/BPO.0000000000001174. PMID: 32028470.*
21. *Yu J, Dumaine AM, Poe-Kochert C, Thompson GH, Mistovich RJ. Seromas Following Pediatric Spinal Deformity Surgery: Is Operative Management Necessary? J Pediatr Orthop. 2020 Apr;40(4):e277-e282. doi: 10.1097/BPO.0000000000001496. PMID: 31876697.*
22. *Makarewich CA, Stotts AK, Yoo M, Nelson RE, Rothberg DL. Inpatient Versus Outpatient Treatment of Gartland Type II Supracondylar Humerus Fractures: A Cost and Safety Comparison. J Pediatr Orthop. 2020 May/Jun;40(5):211-217. doi: 10.1097/BPO.0000000000001442. PMID: 31415017; PMCID: PMC8722678.*
23. *Gomez JA, Kubat O, Tovar Castro MA, Hanstein R, Flynn T, Lafage V, Hurry JK, Soroceanu A, Schwab F, Skaggs DL, El-Hawary R; Pediatric Spine Study Group (PSSG). The Effect of Spinopelvic Parameters on the Development of Proximal Junctional Kyphosis in Early Onset: Mean 4.5-Year Follow-up. J Pediatr Orthop. 2020 Jul;40(6):261-266. doi: 10.1097/BPO.0000000000001516. PMID: 32501899.*
24. *Gardner ROE, Worku N, Nunn TR, Zerfu TT, Kassahun ME. Management of Neglected Traumatic Hip Dislocation in Children. J Pediatr Orthop. 2020 Aug;40(7):e554-e559. doi: 10.1097/BPO.0000000000001535. PMID: 32080056.*
25. *Green C, Brown K, Caine H, Dieckmann RJ, Rathjen KE. Prospective Comparison of Patient-selected Operative Versus Nonoperative Treatment of Scheuermann Kyphosis. J Pediatr Orthop. 2020 Sep;40(8):e716-e719. doi: 10.1097/BPO.0000000000001576. PMID: 32341242.*
26. *Long JT, Laron D, Garcia MC, McCarthy JJ. Screw Anterior Distal Femoral Hemiepiphysiodesis in Children With Cerebral Palsy and Knee Flexion Contractures: A Retrospective Case-control Study. J Pediatr Orthop. 2020 Oct;40(9):e873-e879. doi: 10.1097/BPO.0000000000001634. PMID: 32658158.*
27. *Masquijo JJ, Allende V, Ferreyra A, Hernández Bueno JC. Distal Femoral Physeal Bar Resection Combined With Guided Growth for the Treatment of Angular Limb Deformity Associated With Growth Arrest: A Preliminary Report. J Pediatr Orthop. 2020 Nov/Dec;40(10):e958-e962. doi: 10.1097/BPO.0000000000001651. PMID: 32773655.*
28. *Busch MT, Perkins CA, Nickel BT, Blizzard DJ, Willimon SC. A Quartet of Elastic Stable Intramedullary Nails for More Challenging Pediatric Femur Fractures. J Pediatr Orthop. 2019 Jan;39(1):e12-e17. doi: 10.1097/BPO.0000000000001273. PMID: 30540656.*
29. *LaMont LE, McIntosh AL, Jo CH, Birch JG, Johnston CE. Recurrence After Surgical Intervention for Infantile Tibia Vara: Assessment of a New Modified Classification. J Pediatr Orthop. 2019 Feb;39(2):65-70. doi: 10.1097/BPO.0000000000000933. PMID: 28234732.*
30. *Reddy M, Ho CA. Comparison of Percutaneous Reduction and Pin Fixation in Acute and Chronic Pediatric Mallet Fractures. J Pediatr Orthop. 2019 Mar;39(3):146-152. doi: 10.1097/BPO.0000000000000896. PMID: 30730419.*

31. *Dittmer AJ, Molina D 4th, Jacobs CA, Walker J, Muchow RD. Pediatric Forearm Fractures Are Effectively Immobilized With a Sugar-Tong Splint Following Closed Reduction. J Pediatr Orthop. 2019 Apr;39(4):e245-e247. doi: 10.1097/BPO.0000000000001291. PMID: 30839473.*
32. *Davids JR, Cung NQ, Sattler K, Boakes JL, Bagley AM. Quantitative Assessment of Muscle Strength Following "Slow" Surgical Lengthening of the Medial Hamstring Muscles in Children With Cerebral Palsy. J Pediatr Orthop. 2019 May/Jun;39(5):e373-e379. doi: 10.1097/BPO.0000000000001313. PMID: 30570590.*
33. *Bayhan IA, Abousamra O, Rogers KJ, Bober MB, Miller F, Mackenzie WG. Valgus Hip Osteotomy in Children With Spondyloepiphyseal Dysplasia Congenita: Midterm Results. J Pediatr Orthop. 2019 Jul;39(6):282-288. doi: 10.1097/BPO.0000000000000945. PMID: 31169747.*
34. *O'Brien AO, Stokes J, Bompadre V, Schmale GA. Concomitant Anterior Cruciate Ligament Reconstruction and Temporary Hemiepiphysiodesis in the Skeletally Immature: A Combined Technique. J Pediatr Orthop. 2019 Aug;39(7):e500-e505. doi: 10.1097/BPO.0000000000001330. PMID: 30628975.*
35. *Davis RL 2nd, Samora WP 3rd, Persinger F, Klingele KE. Treatment of Unstable Versus Stable Slipped Capital Femoral Epiphysis Using the Modified Dunn Procedure. J Pediatr Orthop. 2019 Sep;39(8):411-415. doi: 10.1097/BPO.0000000000000975. PMID: 31393301.*
36. *Ulusaloglu AC, Asma A, Rogers KJ, Bowen JR, Mackenzie WG, Mackenzie WGS. Elongation-Derotation-Flexion Casting Treatment of Early-Onset Progressive Scoliosis in Skeletal Dysplasia. J Pediatr Orthop. 2022 Mar 1;42(3):e229-e233. doi: 10.1097/BPO.0000000000002037. PMID: 34967803.*
37. *Modest JM, Brodeur PG, Lemme NJ, Testa EJ, Gil JA, Cruz AI Jr. Outpatient Operative Management of Pediatric Supracondylar Humerus Fractures: An Analysis of Frequency, Complications, and Cost From 2009 to 2018. J Pediatr Orthop. 2022 Jan 1;42(1):4-9. doi: 10.1097/BPO.0000000000001999. PMID: 34739433.*
38. *Limpaphayom N, Stewart S, Wang L, Liu J, Park TS, Dobbs MB. Functional outcomes after selective dorsal rhizotomy followed by minimally invasive tendon lengthening procedures in children with spastic cerebral palsy. J Pediatr Orthop B. 2020 Jan;29(1):1-8. doi: 10.1097/BPB.0000000000000642. PMID: 31305364.*
39. *Segal D, Cobb L, Little KJ. Fracture obliquity is a predictor for loss of reduction in supracondylar humeral fractures in older children. J Pediatr Orthop B. 2020 Mar;29(2):105-116. doi: 10.1097/BPB.0000000000000636. PMID: 31033871.*
40. *Atalar H, Gunay C, Turanli S, Koktener A. Discrepancy between ultrasonographic and radiographic findings in patients treated for developmental dysplasia of the hip; hip maturation on ultrasonography may not be consistent with radiographic hip maturation. J Pediatr Orthop B. 2020 May;29(3):228-234. doi: 10.1097/BPB.0000000000000654. PMID: 31305360.*
41. *Westberry DE, Davis RB, Binkley-Vance R, Westberry A, Westberry A, Wack LI. In-toeing gait in children with clubfoot and the effect of tibial rotation osteotomy. J Pediatr Orthop B. 2020 Jul;29(4):348-354. doi: 10.1097/BPB.0000000000000688. PMID: 31651746.*
42. *Ran L, Chen H, Pan Y, Lin Q, Canavese F, Chen S. Comparison between the Pavlik harness and the Tübingen hip flexion splint for the early treatment of developmental dysplasia of the hip. J Pediatr Orthop B. 2020 Sep;29(5):424-430. doi: 10.1097/BPB.0000000000000667. PMID: 31503108.*
43. *Abousamra O, Sullivan BT, Shah SA, Yaszay B, Samdani AF, Cahill PJ, Newton PO, Sponseller PD. Do seizures compromise correction maintenance after spinal fusion in cerebral palsy scoliosis? J Pediatr Orthop B. 2020 Nov;29(6):538-541. doi: 10.1097/BPB.0000000000000705. PMID: 31821271.*
44. *Huang Y, Feng G, Liu L, Yang X, Song Y, Zhou C, Wang L, Zhou Z. Posterior hemivertebral resection for upper thoracic congenital scoliosis: be aware of high risk of complications. J Pediatr Orthop B. 2019 Jan;28(1):1-9. doi: 10.1097/BPB.0000000000000538. PMID: 30308554.*
45. *Basques BA, Meadows MC, Grauer JN, Kogan M. Pediatric obesity is associated with short-term risks after pelvic osteotomy. J Pediatr Orthop B. 2019 Mar;28(2):95-99. doi: 10.1097/BPB.0000000000000552. PMID: 30234701.*
46. *Andreacchio A, Alberghina F, Paonessa M, Cravino M, De Rosa V, Canavese F. Tobramycin-impregnated calcium sulfate pellets for the treatment of chronic osteomyelitis in children and adolescents. J Pediatr Orthop B. 2019 May;28(3):189-195. doi: 10.1097/BPB.0000000000000517. PMID: 29851713.*
47. *Pontén E, von Walden F, Lenke-Ekholm C, Zethraeus BM, Eliasson AC. Outcome of hand surgery in children with spasticity - a 9-year follow-up study. J Pediatr Orthop B. 2019 Jul;28(4):301-308. doi: 10.1097/BPB.0000000000000600. PMID: 30768582; PMCID: PMC6553991.*
48. *Haruno LS, Kan JH, Rivlin MJ, Rosenfeld SB, Schallert EK, Zhu H, Shenava VR. Spica MRI predictors for epiphyseal osteonecrosis after closed reduction treatment of dysplasia of the hip. J Pediatr Orthop B. 2019 Sep;28(5):424-429. doi: 10.1097/BPB.0000000000000606. PMID: 30807510.*
49. *Ranjan R, Sud A, Adhikary D, Sinha A, Chand S. Incidence and risk factors for iatrogenic distal tibia/fibula fracture during Ponseti technique of clubfoot treatment. J Pediatr Orthop B. 2019 Nov;28(6):572-578. doi: 10.1097/BPB.0000000000000595. PMID: 30741748.*

50. *Kadhim M, Gauthier L, Logan K, El-Hawary R, Orlik B. Guided growth for angular correction in children: a comparison of two tension band plate designs. J Pediatr Orthop B. 2018 Jan;27(1):1-7. doi: 10.1097/BPB.0000000000000492. PMID: 28799981.*
51. *Farr S, Ganger R, Girsch W. Distal humeral flexion osteotomy for the treatment of supracondylar extension-type malunions in children. J Pediatr Orthop B. 2018 Mar;27(2):115-120. doi: 10.1097/BPB.0000000000000470. PMID: 28628579.*
52. *Lee M, Kelly E, Kelly P. Botulinum-A intramuscular injection in an orthopaedic paediatric patient cohort: service restructure analysis. J Pediatr Orthop B. 2018 May;27(3):189-193. doi: 10.1097/BPB.0000000000000488. PMID: 28704302.*
53. *Flipsen M, Ham JS, van der Zwan AL, Mader K. Radial head resection and hemi-interposition arthroplasty in patients with multiple hereditary exostoses: description of a new surgical technique. J Pediatr Orthop B. 2018 Jul;27(4):289-295. doi: 10.1097/BPB.0000000000000496. PMID: 29023265.*
54. *Song MH, Jang WY, Park MS, Yoo WJ, Choi IH, Cho TJ. Slipped capital femoral epiphysis in children younger than 10 years old: clinical characteristics and efficacy of physeal-sparing procedures. J Pediatr Orthop B. 2018 Sep;27(5):379-386. doi: 10.1097/BPB.0000000000000485. PMID: 28704299.*
55. *Georgiadis AG, Dutt V, Truong WH, Novotny SA, Novacheck TF. Anteverting Bernese periacetabular osteotomy in the treatment of neurogenic hip dysplasia in cerebral palsy. J Pediatr Orthop B. 2018 Nov;27(6):473-478. doi: 10.1097/BPB.0000000000000513. PMID: 29708908.*
